# Smart molecules for imaging, sensing and health (SMITH)

**DOI:** 10.3762/bjoc.11.274

**Published:** 2015-12-10

**Authors:** Bradley D Smith

**Affiliations:** 1 University of Notre Dame, Notre Dame, IN 46530, USA

**Keywords:** fluorescence, ion-pair receptors, membrane transport, molecular imaging, rotaxane, squaraine

## Abstract

This autobiographical review provides a personal account of the author’s academic journey in supramolecular chemistry, including brief summaries of research efforts in membrane transport, molecular imaging, ion-pair receptors, rotaxane synthesis, squaraine rotaxanes, and synthtavidin technology. The article concludes with a short perspective of likely future directions in biomedical supramolecular chemistry.

## Review

### From the land of kangaroos to the home of the Fighting Irish

**Figure 1:**
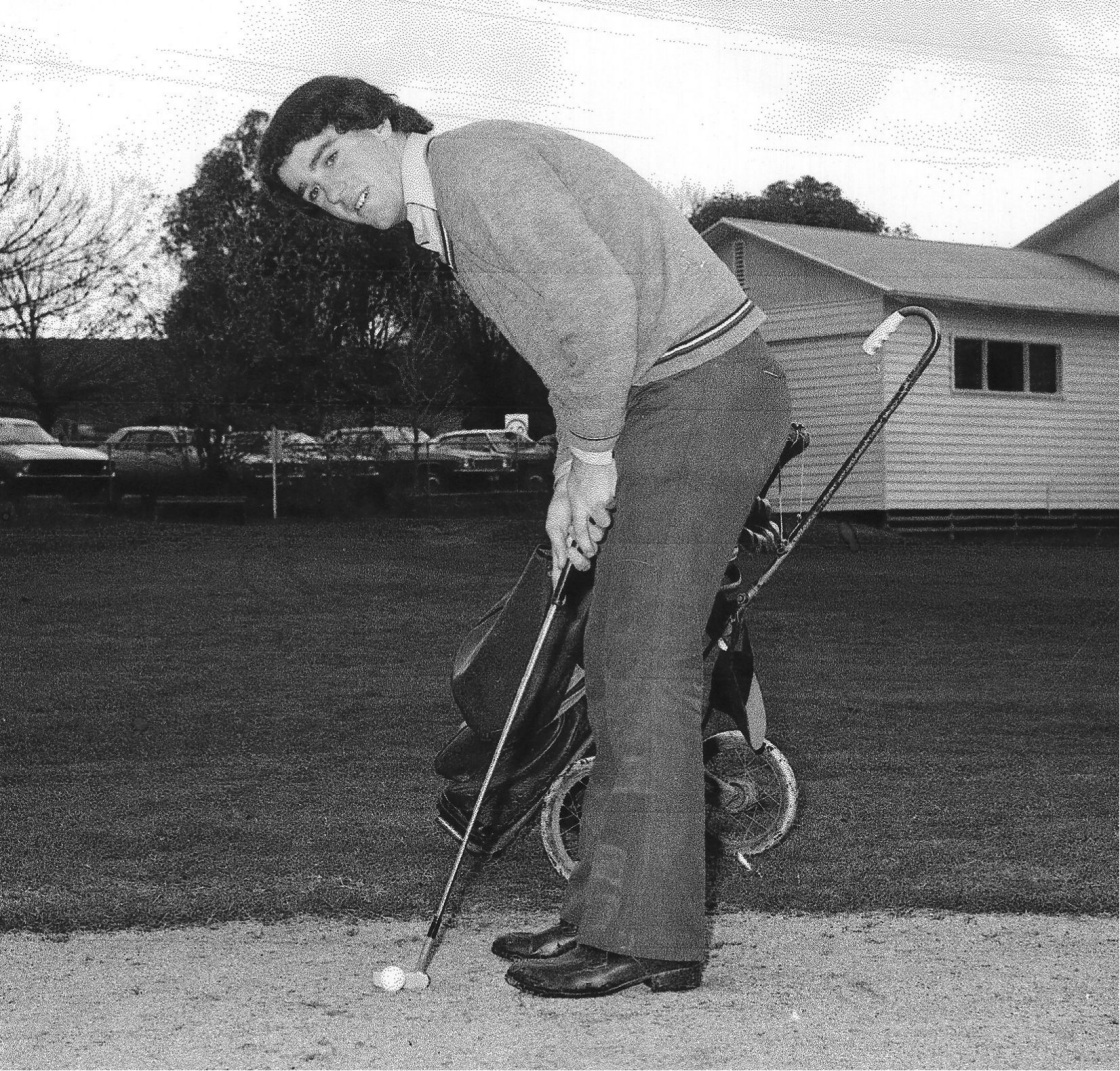
The author as a teenager in his school uniform, but on the nearby Myrtleford golf course.

I grew up in Myrtleford, a small town in the foothills of the Australian alpine region (Australia has mountains?). The town population of ≈2,500 has hardly changed in fifty years, and I return there regularly to visit my parents. I attended the local schools and although I was clearly one of the smarter students, a recent review of written teacher comments reveals a propensity to be the class clown. Most of my teenage years were spent on the local golf course (Figure 1), but I was sufficiently successful in the statewide exams to gain entry to the University of Melbourne as an undergraduate science major. Mediocre performances in maths and physics soon left chemistry as the only viable option for serious advancement, and it was in my third year that I encountered my first research experience under the supervision of Dr. Roger Read (now at the University of New South Wales). The project goal was modest but I enjoyed the personal ownership and also the camaraderie of working in a large open lab. I was sufficiently successful to get accepted into a fourth year of study for B. Sc. Honors, and I joined the group of Dr. David Kelly and worked on an NMR project on the structure of carbocations. I was lucky enough to stumble upon an unusual naphthalenium rearrangement process and I worked hard enough to earn co-authorship of a full paper in the Journal of the American Chemical Society [1]. As the year progressed I began to think about future plans. It was very common for Australian college graduates to head overseas on lengthy world trips, but I greatly enjoyed my research experience, so a reasonable compromise was graduate studies abroad. I met with Dr. Kelly for suggestions and the outcome was a plan to join the lab of his former mentor, Professor Lloyd Jackman, an Australian who had moved from Melbourne to Penn State University in the United States. Professor Jackman was a pioneer in the use of NMR methods for studies of organic chemistry, and my graduate project was to elucidate the aggregated structures of lithium enolates in a weakly polar solvents using a range of multinuclear NMR methods [2]. The work involved vacuum line preparation of the samples and extensive measurements of NMR parameters. Jackman was a wonderful academic advisor, a passionate scientist who also enjoyed sports and recreation. He was beloved by his students and respected by his colleagues as a scholar and a gentleman. A quote that I sometimes attribute to Jackman but it may in fact predate him is, “A physical organic effect has to produce more than a 1000-fold difference to be truly interesting”. Upon graduation I moved to Oxford University in the UK for postdoc studies in the group of legendary Professor Jack Baldwin, at the height of his work on penicillin biosynthesis. The lab was full of very talented people, but the enormous size of the group made it unwieldy. After 15 months and a few quick papers I moved back to the US for a second postdoc with Professor Koji Nakanishi at Columbia University in New York City. Professor Nakanishi was a larger than life character, who formed strong bonds with his students and postdocs. He worked very long hours in an office that was open to the labs and often joined his students for meals or late evening drinks. He was a natural showman and dazzled his lecture audiences with magic tricks and engaging stories. The lab worked on an array of technically challenging projects concerning natural products isolation and their mode of action. Nakanishi worked closely with several pharmaceutical companies and it was inspiring to see the immense resources that large companies could focus on important pharmaceutically projects. So when I started looking for a permanent job, I was initially torn between academics and industry. But my interviews at universities seemed to progress more smoothly and in 1991 I accepted an attractive offer to start a tenure-track position in the Department of Chemistry and Biochemistry at the University of Dame, the home of the Fighting Irish.

My Ph.D. and postdoc training covered a wide range of physical organic and bio-organic topics but none of them could be considered supramolecular chemistry. So it was a little risky to start an independent academic career in a field with no previous experience. Indeed my new lab had to start from the beginning and learn all the rudimentary supramolecular experimental techniques such as measuring binding constants and conducting transport experiments. The lack of background knowledge was an obvious impediment, but at times it was helpful because I was not biased to revisit old supramolecular problems from the previous decade. As I look back at my independent research publications, it seems that the more highly cited papers were often projects that required the lab to learn a new experimental technique or move into a completely new research area. So while the learning curve was steep, the eventual reward was a more impactful outcome.

### Major research projects

**Scheme 1:**
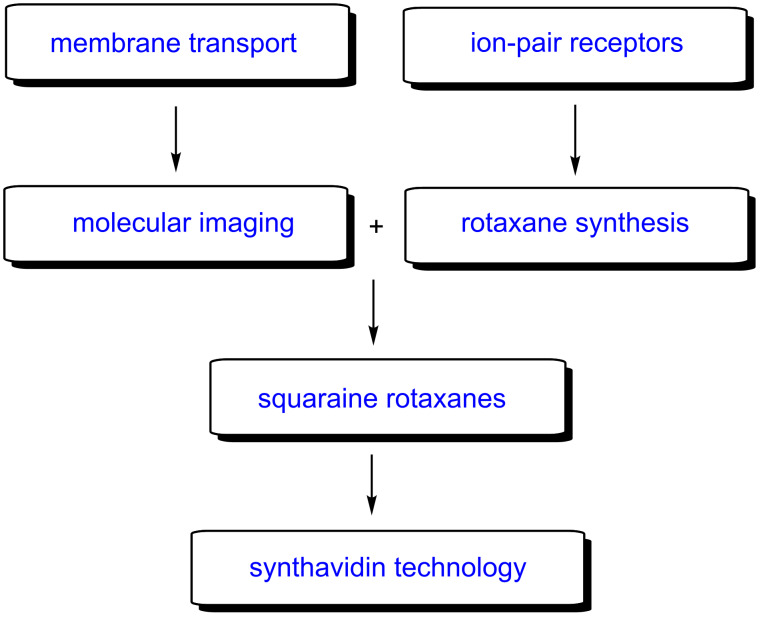
Chronological progression of Smith group research projects.

In Scheme 1 is a flow diagram of major topics that my research group has pursued over the last 25 years. I maintain a group of about ten full-time co-workers, along with a handful of undergraduate researchers, and at any time about two thirds of the co-workers are organic chemists and the rest are biochemists. Most of the projects have started out as structure design problems where the goal was to prepare a set of organic molecules with specific supramolecular functions. Once the molecules were made they had to be tested and in the early days this typically involved simple test-tube studies, but increasingly the testing systems became more biological and more relevant to human health. This required us to collaborate with an expanding number of research specialists. Collaboration can be highly rewarding and without doubt it is the best way to maintain an internationally competitive program in modern supramolecular chemistry. But young investigators must quickly recognize that collaboration is an exercise in human relationships, and it works best when there is obvious benefit to both parties and strong lines of communication.

The following sections expand on Scheme 1 and provide short summaries of the major research projects. The main purpose is to illustrate the chronological flow of thoughts and events that led me to pursue a series of supramolecular ideas that may seem from the outside to be disconnected. Since this article is highly autobiographical, it does not cite all of the seminal and inspirational work done by other research groups, and I apologize for any distortion of credit. To assist the interested reader, each research topic includes references to relevant review articles by leading groups in the field.

### Membrane transport

**Scheme 2:**
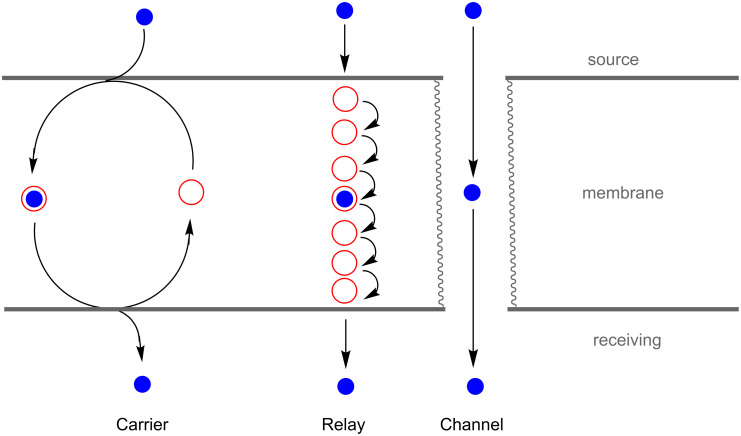
Molecular transporters promote translocation of ions or hydrophilic biomolecules across a synthetic or biological membrane.

Molecular transporters that alter the concentration gradients of anions and biomolecules across a cell membrane have various biological effects as reagents for cell biology research and as potential pharmaceuticals [3–4]. In the early 1990’s, most of the supramolecular research on facilitated membrane transport using carrier molecules focused on metal cation transport. As a young professor, with an inexperienced group, we started out looking at synthetic transport carriers for biomolecules such as sugars, nucleotides, amino acids, and catecholamines [5] (Scheme 2). Later on we started looking at chloride transport and we enjoyed a very productive collaboration with the British group of A. P. Davis to develop a series of mobile carriers for chloride ions across liposome and cell membranes [6]. There continues to be a strong community interest in chloride transporters as they are expected to exhibit interesting biological activity [7]. In addition, my group developed some of the first synthetic molecules to promote flip-flop of phospholipids across cell membranes, and thus alter the membrane structure and function. In particular, we discovered compounds that can scramble the transmembrane distribution of phosphatidylserine, a crucial signaling phospholipid. The synthetic scramblases make phosphatidylserine appear on the surface of cells and induce subsequent secondary biological signaling processes such as blood clotting and cell clearance by macrophages [8].

### Molecular imaging

**Figure 2:**
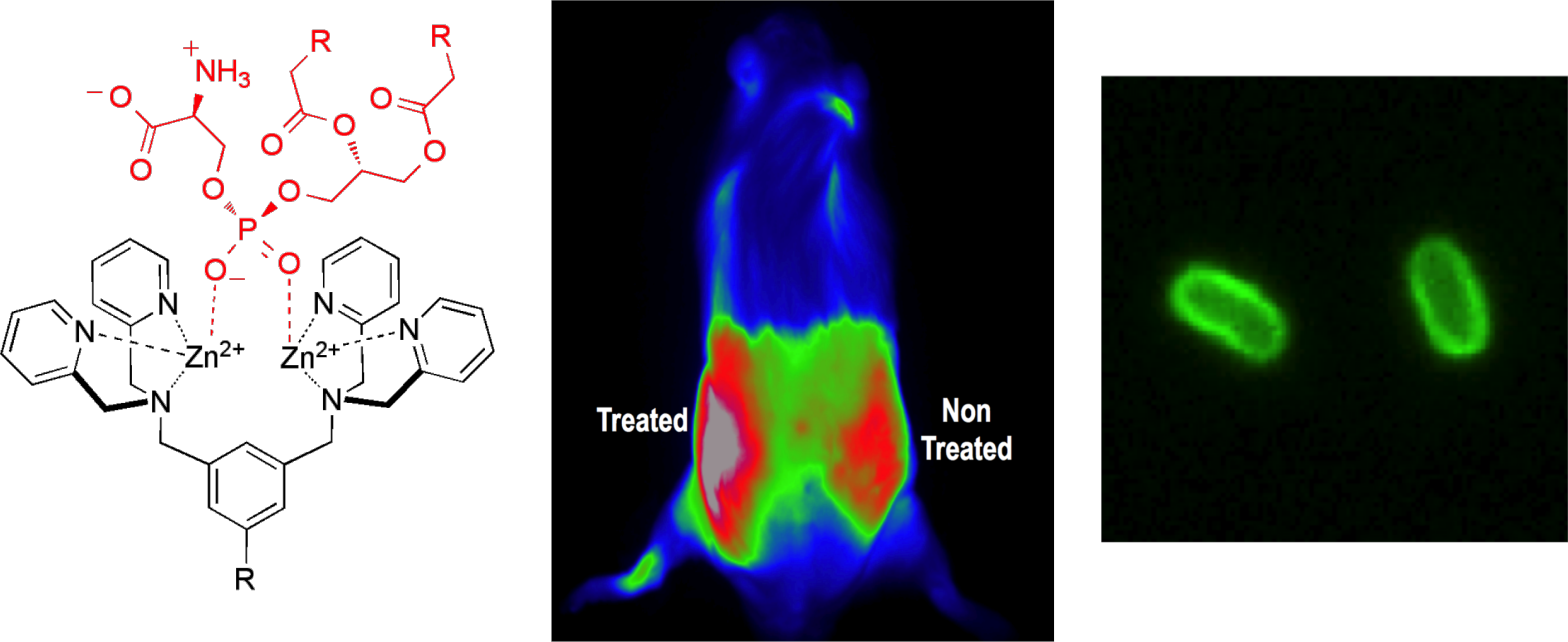
(left) Association of ZnDPA probe with phosphatidylserine head group. (middle) False colored fluorescence image of a living rat bearing two tumors. There is increased accumulation of the cell death probe in the tumor that has been treated by focal beam radiation. (right) Fluorescence image of bacterial cells stained with fluorescent ZnDPA probe.

Our work on synthetic phospholipid scramblases required us to develop methods for quantifying phospholipid levels on the surface of biological membranes [9–10]. Biomembrane science is very challenging because the self-assembled bilayer is a soft and dynamic object that is hard to characterize with spectroscopic methods. To paraphrase a former US president, “we pursue biomembrane science not because it is easy but because it is hard” [11]. We realized that most of the targeted fluorescent molecular probes for phospholipids were derivatives of large protein systems and we felt that we could devise small synthetic mimics of the binding pockets. Our primary phospholipid target was phosphatidylserine, which gets exposed on the cell surface during the process of apoptosis or programmed cell death. Thus, phosphatidylserine is an excellent biomarker of cell death and an attractive target for molecular imaging. We reasoned that synthetic anion receptors such as zinc dipicolylamine (ZnDPA) coordination complexes would selectively recognize the anionic head group of phosphatidylserine [12]. Furthermore, binding strength is amplified by electrostatic attraction of the cationic receptor to the apoptotic cell membrane with a negative surface potential. We developed a family of fluorescent ZnDPA probes for fluorescence microscopy and flow cytometry methods for preclinical research [13–16] (Figure 2). Many of these fluorescent probes are used as imaging reagents to quantify the level of cell death in a range of biomedical samples [17]. We also developed some nuclear isotopic labeled probes for in vivo imaging of living subjects [18]. An eventual goal of this ongoing research project is to invent clinical imaging methods that will improve patient care by rapidly evaluating the efficacy of cancer treatment or the extent of cardiovascular disease.

The capability of these ZnDPA probes to target anionic cell surfaces led us to pursue molecular imaging of microbial infection, an unsolved research problem of high biomedical significance. We demonstrated targeted optical imaging of bacterial infection in animal models and subsequently developed optical probes for photodynamic therapy of bacterial infection [19–22]. The latest discovery is a set of molecular ZnDPA probes that selectively target parasite infections in living subjects such as Leishmaniasis, a lethal disease that afflicts many millions of people around the world [23].

### Ion-pair receptors

**Scheme 3:**
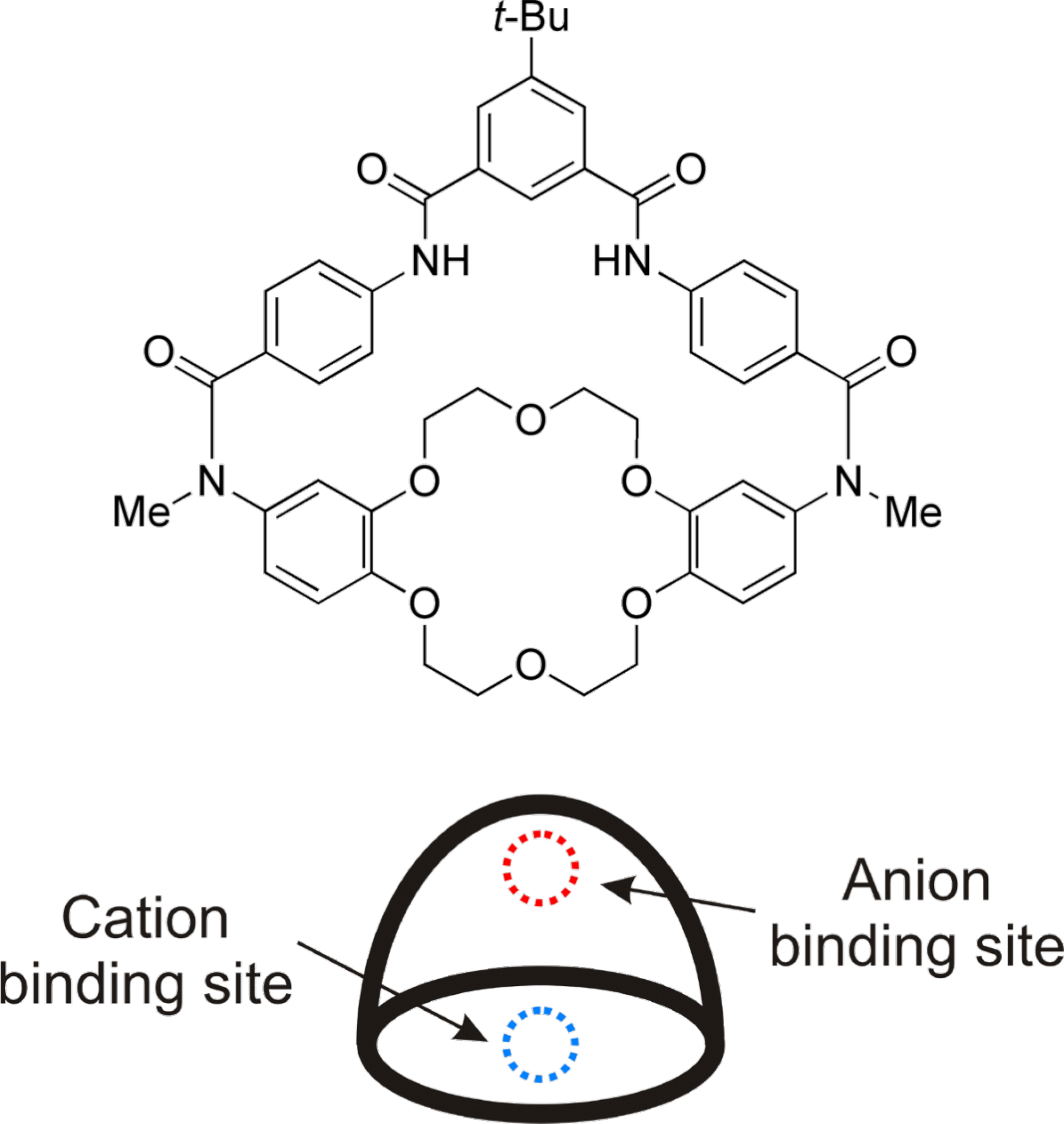
Macrocyclic receptor that binds solvent separated ion-pairs.

Our early experience with membrane transport raised awareness of the need for transport carrier molecules that could simultaneously complex both the anion and the cation [24–25]. Starting in 2000 we prepared a number of relatively simple macrobicyclic receptors that could bind salts as either contact ion-pairs or as solvent separated ion-pairs [26–28] (Scheme 3). Many X-ray crystal structures were acquired and they provided excellent atomic scale insight. The liquid extraction and membrane transport properties were evaluated and we demonstrated symport processes that used anion concentration gradients as energy sources to drive cation transport uphill [29].

### Rotaxane syntheses

**Scheme 4:**
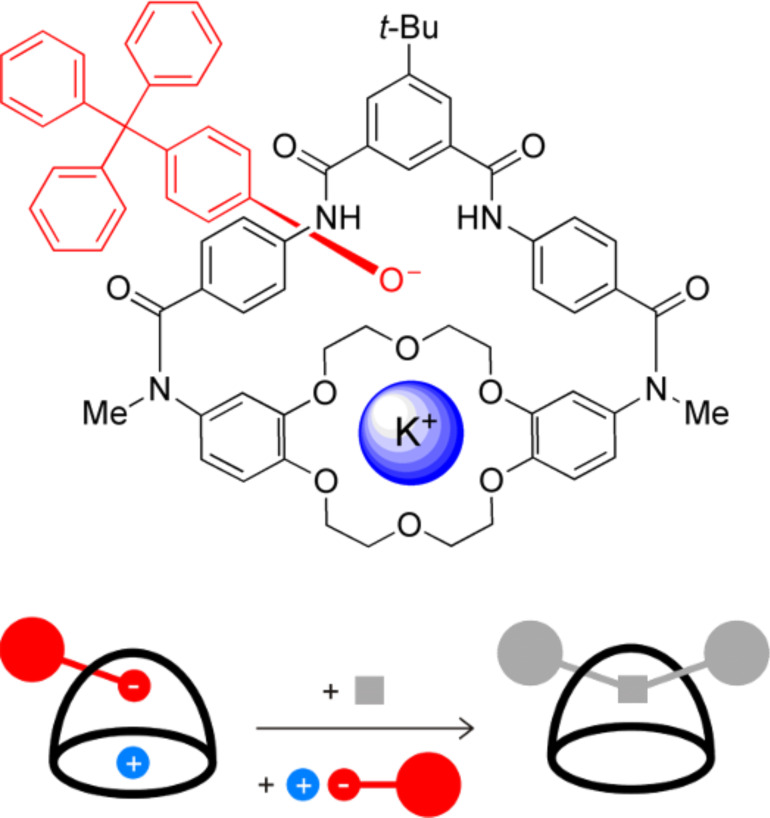
Trapping a macrocyclic receptor containing a reactive ion-pair produces an interlocked [2]rotaxane.

Building on literature ideas about “wheeled nucleophiles [30–31].” we prepared the reactive ion-pair in Scheme 4 and trapped the bound phenolate as an uncharged [2]rotaxane structure [32]. The ability of the macrobicyclic receptor to accommodate a solvent separated ion-pair was vital for efficient rotaxane formation. The interlocked molecule retained its salt binding ability and association of the ions modulated the rotaxane structural dynamics [33–34].

### Squaraine rotaxanes

**Figure 3:**
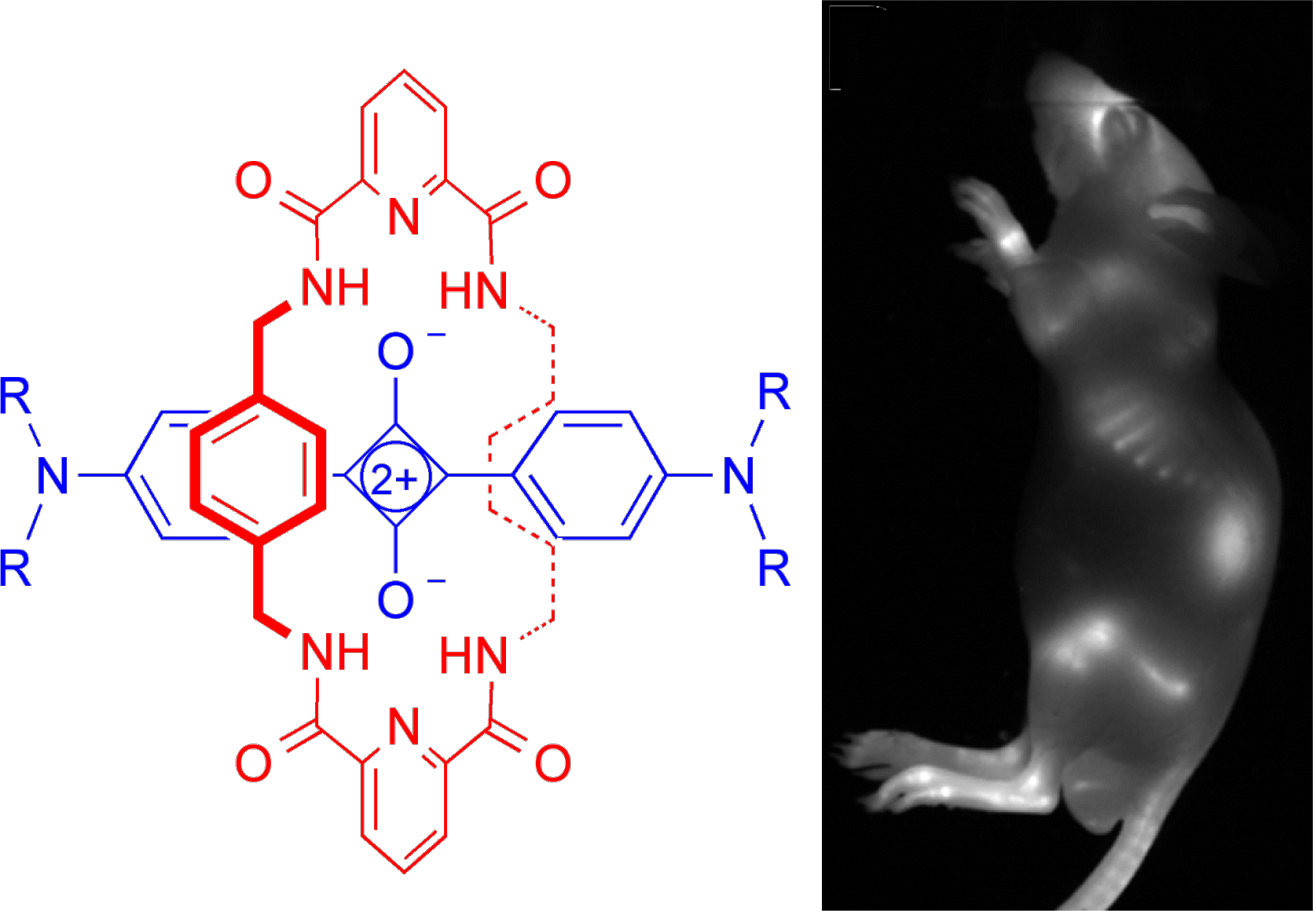
(left) General structure of a squaraine rotaxane dye. (right) Fluorescence image of a living mouse dosed with a squaraine rotaxane probe that selectively targets bone. Reprinted with permission from [37]. Copyright (2013) American Chemical Society.

The interests in molecular imaging and rotaxane structures merged in 2005 with the discovery and development of squaraine rotaxanes as a novel family of deep-red fluorescent dyes with extremely high brightness and stability [35–36]. A key finding was the importance of the interlocked rotaxane structure for protecting the encapsulated squaraine from chemical attack by water. Squaraine rotaxanes can be conjugated with targeting ligands, and bioimaging studies have shown that they enable fluorescence microscopy and mesoscale imaging of diverse biomedical targets such as tumors, infection, bone, cell death, and brown adipose tissue [37–38] (Figure 3). Several of these molecular probes are commercially available for preclinical research applications including acceleration of the anticancer and obesity drug discovery, facile monitoring of bone growth, and next-generation fluorescence-guided surgery [17]. An off-shoot of this work was discovery of novel chemiluminescent squaraine rotaxane structures [39]. Nanoparticles containing these self-illuminating molecules enable high sensitivity imaging of deep-tissue target sites in living subjects [40].

A recent spin-off from the squaraine rotaxane project uses homologous croconaine dyes to absorb 800 nm laser light and cleanly convert the energy into heat without producing reactive singlet oxygen [41–43]. The dyes enable new types of nanoscale heating technologies that release sensitive payload such as dyes, drugs, oligonucleotides, or proteins. The dyes can also be loaded into nanoparticles for anticancer photothermal therapy in preclinical animal models. Another use for these dyes is as contrast agents for photoacoustic imaging where short laser pulses are converted into sound waves that are detected using an ultrasound scanner.

### Synthavidin technology

**Scheme 5:**
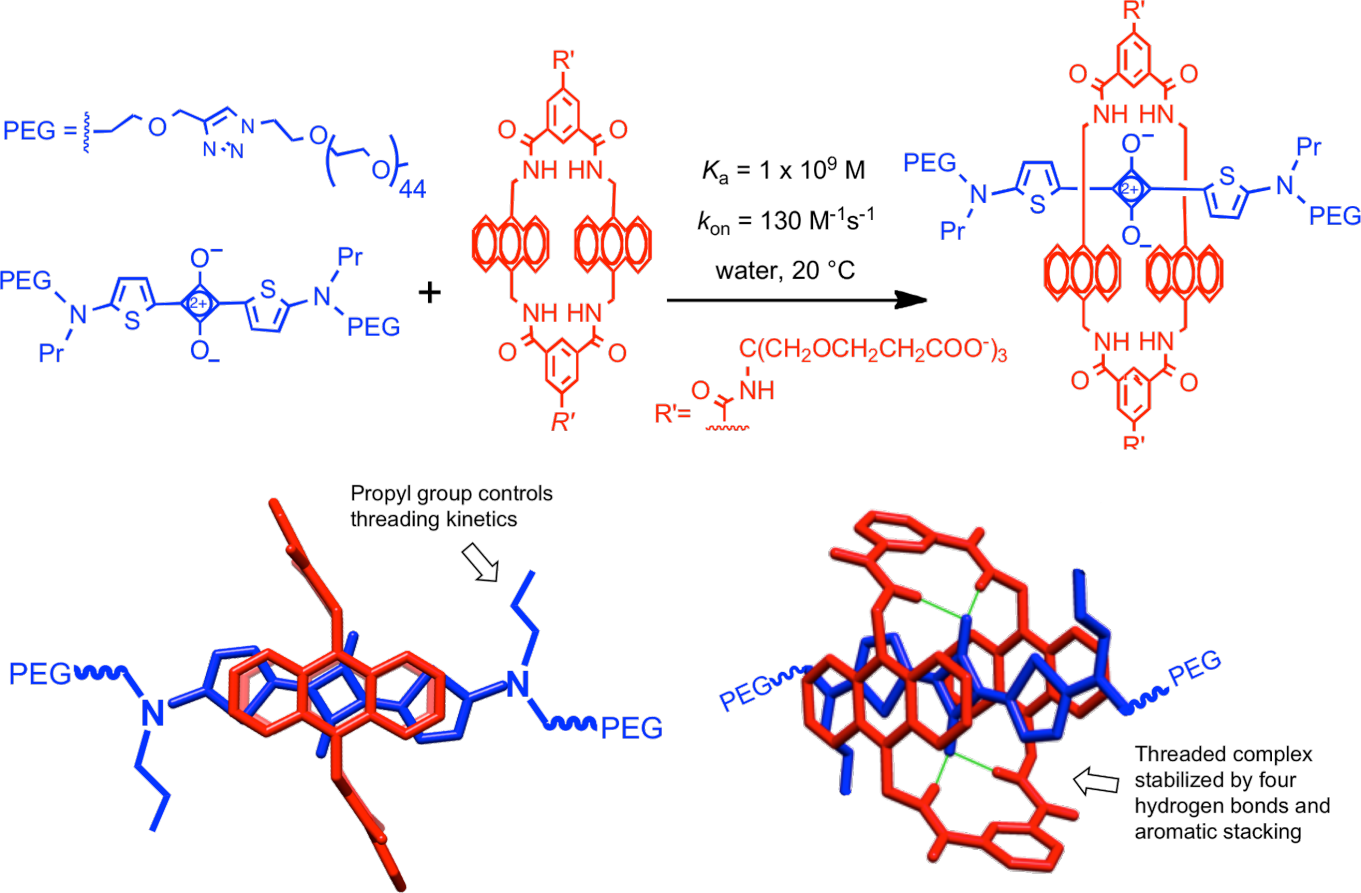
(top) Basis of Synthavidin technology. A fluorescent squaraine dye that is flanked by PEG chains can rapidly thread a tetralactam macrocycle in water to produce a highly stable complex. (bottom) Two views of the threaded complex highlighting the structural features controlling the threading kinetics and thermodynamics.

While working with the molecular building blocks to make squaraine and croconaine rotaxanes we discovered a remarkable self-assembly process that we call Synthavidin (synthetic avidin). A simple example is illustrated in Scheme 5 [44]. A water soluble tetralactam macrocycle with anthracene sidewalls is threaded by squaraine dyes that are flanked by long PEG chains to give highly stable complexes with nanomolar dissociation constants. The rate of macrocycle threading is insensitive to the length of the appended PEG chains. But the threading kinetics are greatly affected by the steric size of the second *N*-substituent at each end of the squaraine dye, and an *N*-propyl group produces a perfect mixture of kinetic and thermodynamic properties. The nanomolar affinity in water is exceptionally strong for a synthetic cyclophane host molecule, and it is driven by a large favorable change in enthalpy. The two oxygen atoms on the encapsulated squaraine dye form hydrogen bonds to the four macrocycle NH residues and there is coplanar stacking of the squaraine aromatic surfaces with the anthracene sidewalls of the macrocycle. We are beginning to use Synthavidin technology as a self-assembly platform to rapidly fabricate libraries of targeted multivalent probes for fluorescence imaging of over-expressed receptors on the surface of cancer cells. We are hopeful that the probes will be useful for therapeutic applications such as fluorescence-guided surgery and intra-operative photodynamic therapy.

### Life as an academic supramolecular chemist

This is my 25th year at the University of Notre Dame, which is located in South Bend, Indiana, 90 miles from Chicago. My wife’s parents live a few hours away and my two daughters are presently undergraduates at the university. Typically, our family holidays are spent within Indiana or we travel all the way to Australia. Travel is an integral part of academic research, and it is always enjoyable to visit a new part of the world. I have little ability to learn new languages and I am very grateful that English is the universal language of science. I can hardly imagine the challenge of writing papers and presenting lectures in a non-native language. In the summer time, my primary source of recreation is golf or cycling. In the Fall, many weekends are filled with the social aspects of college football. It may hard for non-American readers to imagine tail-gate parties in the campus parking lots with tens of thousands of like-minded fans. But college football is essentially the university’s social season and it provides a great backdrop for hosting visitors to the campus.

**Figure 4:**
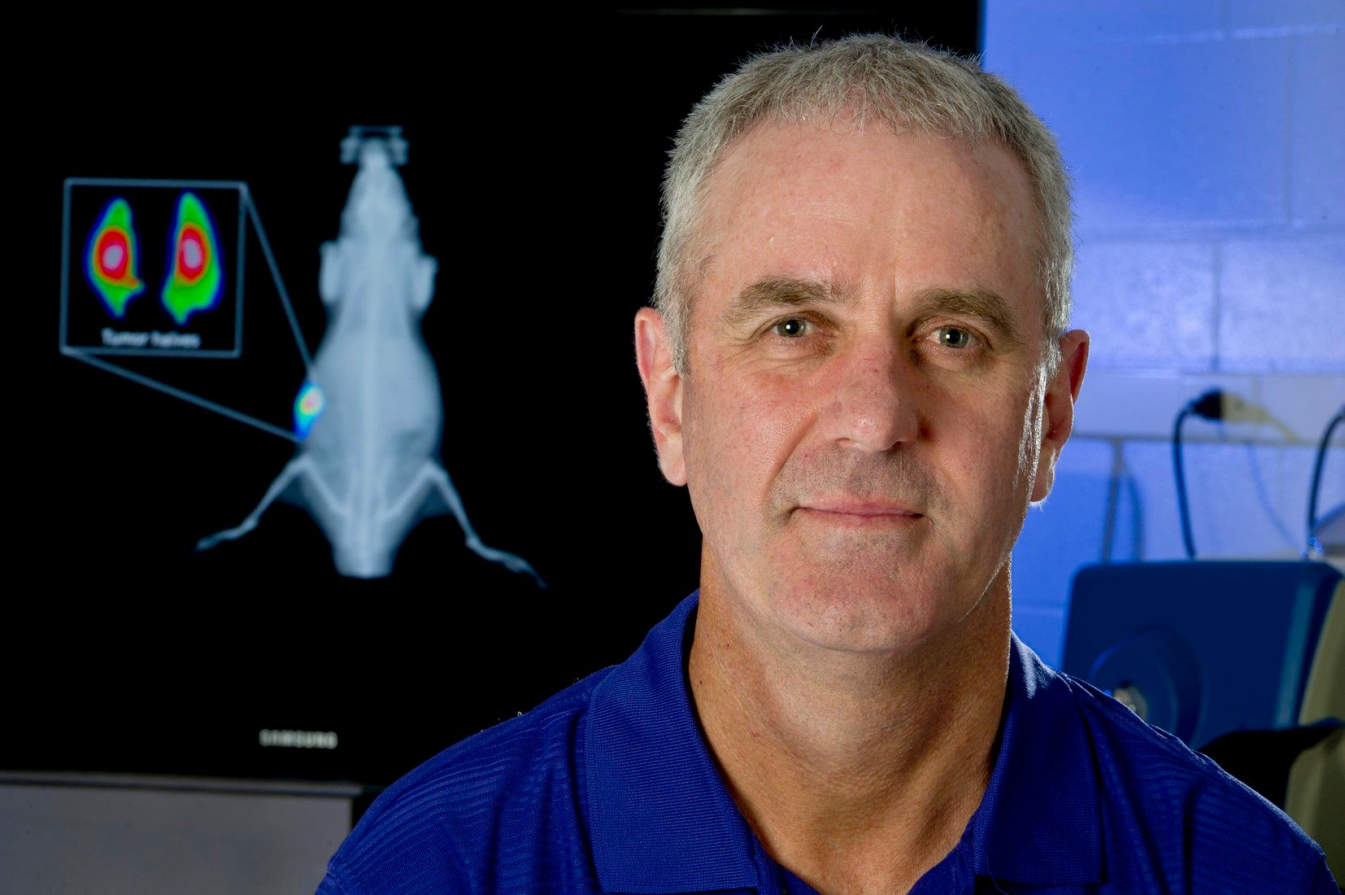
The author as director of the Notre Dame Integrated Imaging Facility.

A trend that often occurs over the span of an academic career is a change in the primary motivation for conducting research. The goal of most young investigators is to achieve a list of well-defined accomplishments (obtain a major grant, publish high impact papers, get promoted, speak at major conferences, etc.) but for senior investigators there is often a growing desire to make a broader contribution that has lasting impact. This contribution could be in any of the three major components of academic life, namely, research, teaching, or administration. It is my observation that chemists often are well-suited for administration, most likely because they have experience managing relatively large research groups. In my own case, I serve my university as Director of the Notre Dame Integrated Imaging Facility, a campus wide research core that houses about a dozen major instruments that are maintained by seven staff members (Figure 4). It has been very rewarding to create this facility and see it grow to help the broader research effort of the university. Of course, the most obvious way to make a significant contribution to academic research is to publish high impact papers, and there is tremendous gratification when other investigators employ ideas or techniques that were first developed in your own lab. A tangible outcome of our lab is production of new fluorescent molecular probes and I am proud that more than dozen of our probes are commercially available and used by researchers around the world [17].

The US is an exciting place to conduct academic research, especially for a young investigator since there is opportunity to quickly build a large independent research group. But the US granting system has strong Darwinian elements and there is always pressure to procure research funding. The grant writing process can sometimes be tiresome, but it also can be enlightening. As a reminder to always view the glass as half-full, I have the following quotation posted in my office; “In academics, the situation is often desperate but never very serious”. In other words, I am a tenured professor who has the privilege of spending every day with bright young students who have open minds and a desire to learn. My job is to guide them and to channel their optimism into fulfilled potential. There is no doubt that at career-end, my greatest overall contribution will be the outstanding group of young scientists who have trained in my lab and gone on to make important contributions to their families, communities, and broader society.

### Future directions in biomedical supramolecular chemistry

Recently, I co-authored a book chapter entitled “Applications of Synthetic Receptors for Biomolecules [45].” The chapter grouped the most common applications into four general groups; Separations, Imaging and Sensing, Catalysis, and Pharmaceutical Activity – important topics that will continue to attract the attention of many supramolecular chemistry labs around the world. In addition, the chapter provided a short summary of four emerging themes in biomolecule recognition that are likely to garner increased attention over the next decade; Logic Devices, Biomolecule Responsive Materials, Drug Delivery, and Biomolecule-Fueled Molecular Machines. While there are presently “proof of concept” studies for each of these research themes [46–47], I feel there is a need to advance beyond prototype systems. If supramolecular chemistry is to maintain its legitimacy as a field worthy of major academic and industrial investment, it has to clearly show a pathway that leads to valuable new technologies.

Effective biomolecule recognition requires both strong affinity and high selectivity, and for investigators who wish to develop association systems that operate effectively in water I offer two words of advice “surface area”. In 2003, Houk and coworkers published a survey of all known natural and synthetic host/guest binding systems and concluded that the best predictor of strong affinity is the amount of solvent accessible surface area that is buried upon binding [48]. Typically, this surface area is amphiphilic; that is, it contains a mixture of polar and non-polar functional groups. In water, the non-polar groups drive affinity due to hydrophobic effects, and the polar groups produce selectivity due to directional interactions, such as hydrogen bonding. It is insightful to consider the protein–protein recognition and self-assembly processes that are crucial for cell growth and signaling. These precisely controlled association systems have been refined over billions of years of evolution. The selective recognition is driven by protein–protein interactions that operate at an interface with a surface area that is usually in the range of 1200 to 2000 Å^2^. This is more than an order of magnitude greater than the surface area of the largest synthetic molecular hosts, and I think there is an important underlying message here – to achieve biologically relevant binding systems with truly useful dynamic properties, the supramolecular community should build much larger synthetic hosts. How can this be done? One approach is to create covalently linked polymers that are programmed to fold up into three dimensional structures that mimic the topologies of proteins and nucleic acids. Perhaps a more facile fabrication strategy is to employ hierarchal self-assembly methods to create robust nanoscale architectures with precisely positioned functional groups. I am attracted to this latter strategy for enhanced biomolecule recognition and indeed our emerging efforts with Synthavidin technology are small steps in the general direction. This vision merges classical supramolecular chemistry with materials self-assembly. The projects will be technically challenging, as they will require nanoscale characterization methods that are not familiar to most small molecule chemists. Thus, collaboration is likely to be a key element for this type of multiscale work. Intellectually, the investigators need to broad minded and willing to learn the language and culture of other research fields. Early success is not guaranteed but the journey will be fascinating.
